# Dietary Habits and Nutritional Challenges of the Elderly in Ghana

**DOI:** 10.1155/2023/3011067

**Published:** 2023-02-25

**Authors:** Auswell Amfo-Antiri, Nana Ama Frimpomaa Agyapong, Linda Cobbah

**Affiliations:** ^1^Department of Integrated Home Economics Education, Faculty of Home Economics, University of Education, Winneba, Ghana; ^2^Department of Clinical Nutrition and Dietetics, College of Health and Allied Sciences, University of Cape Coast, Cape Coast, Ghana

## Abstract

The elderly population is increasing worldwide. Dietary habits play a crucial role in prolonging life and preventing diseases. This cross-sectional study sought to investigate the dietary habits of the elderly in the Kwahu South District of the Eastern Region of Ghana and further ascertain the factors that constitute nutritional challenges among this group. A mixed method approach was used for the study. A questionnaire and focus group discussion guide were used to solicit data from study participants. A total of 97 participants made up of 59 males and 38 females participated in the study. Data on food habits reveal that staple food consumption especially those grown within the study area is common. Rice (34.1%), game meat (47.1%), banana (63.9%), and garden eggs (27.8%) were the commonly consumed foods by frequency. Mood (41.2%) and stress (24.8%) were identified as the most predominant determinants of food habits. Poly medication, toothache and loss, immobility, and financial and technological challenges were amongst the nutritional challenges mentioned by the elderly in this study. Results from the focus group discussion revealed high nutrition knowledge among the elderly through factors such as financial constraints that were mentioned as a barrier to the translation of this knowledge into practice. Strengthening of existing interventional programmes such as the Livelihood Empowerment Against Poverty and social interventions is needed to improve the dietary habits and nutritional intakes of the elderly.

## 1. Introduction

The elderly population is increasing around the globe. Data from the World Health Organization indicate that the proportion of the global population who are 60 years and older is 1 billion—the figure is projected to reach 1.4 billion by the year 2030 [1]. This is attributed to improved healthcare and food systems worldwide, leading to a reduction in fertility, infant and maternal mortality, improved nutrition, and a reduction in infectious and parasitic diseases [2, 3]. The rapidly aging population is a phenomenon mostly known in developed countries, but currently, low- and middle-income countries are experiencing the greatest increase in their aging population [4]. Japan already has about 30% of its population above the age of 60 years [5]. The World Health Organization predicts that by 2050, two-thirds of the world's population who are 60 years and above will live in low- and middle-income countries such as Ghana [1]. Trend analysis by Mba [6] shows that the aged population increased exponentially between 2000 and 2006. Mba [6] further projects that if this trend continues, over 2 billion older persons will be alive by 2050.

In Ghana, there has been a consistent increase in the number of older persons and the country has one of the highest percentages of the older population in sub-Saharan Africa. The proportion of the elderly population increased from 4.9% in 1960 to 7.2% in 2000 [6]. There is evidence to suggest that, the older population in Ghana are living longer in recent years with about 976,000 of the population documented to be aged 65 years or older in 2020 [7]. Estimates from the 2021 Ghana population and housing census indicate that 4.3% of the Ghanaian population is 65 years or older [8].

Aging as a gradual and continuous process is characterized by a natural gradual decline in bodily functions of a person in early adulthood [9]. This decline in health and function is further exacerbated by loss or a significant reduction in income levels and reduced mobility which has the potential to affect the attainment of good health care and optimal nutrition [9-12]. Optimal nutrition plays an essential role in averting a lot of age-related diseases and improving the quality of life of the aged [13]. The risk of various nutrition-related noncommunicable diseases increases with advancing age [14]. Inflammageing is an underlying cause of several age-related chronic conditions, including stroke, heart diseases, and cancer, all of which can be averted by the intake of nutritious foods [15]. The Mediterranean diet, for instance, is rich in fruits and vegetables, as well as whole grains and antioxidants which has been well documented to decrease chronic disease risks associated with inflammageing [16, 17]. Veronese et al. [18] in a longitudinal study found that the incidence of frailty was twice as much in participants not adhering to the Mediterranean diet compared to those who adhered to it. In older women, phytoestrogens found in soyabeans prevent the occurrence of post-menopausal osteoporosis while lycopene in foods such as tomatoes improves prostate health among men [19–21]. In spite of the critical role nutrition plays in older age, the elderly group is at significant risk of malnutrition and diet-related diseases and faces various nutrition challenges that affect their dietary habits and overall wellbeing [22]. Research conducted among older persons in Ghana by Agbozo et al. [23] showed that greater than 50% of study participants had a poor dietary pattern. In a recent study by Abekah-Carter et al. [24], it was found that adequate nutrition was among the greatest needs expressed by older adults. Studies delving into the dietary habits of older adults and the nutrition challenges they face are scanty in Ghana and more so among older persons in the Eastern region of the country. Such studies are needed to create awareness and advocate for interventions among policy makers and key stakeholders.

This cross-sectionalmixed-method study, therefore, assessed the dietary habits and nutritional challenges of older adults in the Eastern region of Ghana.

## 2. Methods

This was a cross-sectional study in which we employed a mixed-method approach to obtain data on the dietary patterns and nutritional challenges of the elderly. The use of the mixed-method approach was to allow us to support quantitative data obtained with an in-depth analysis of qualitative data. Furthermore, the aim of employing a mixed-method design was to gain credible and valid research findings by using multiple approaches and multiple sources of information to overcome biases [25, 26].

### 2.1. Study Setting

This study was conducted in the Kwahu South District of the Eastern Region of Ghana. The district is amongst the mountainous areas of the Eastern region of the country. The area has a natural environment where subjects typically go about their daily activities, which include but are not limited to agriculture, tourism, pottery, fishing, hunting, and petty trading. Food types in the district primarily encompass various tropical staple food crops (cassava, plantain, cocoyam, yam, taro, and maize), legumes, vegetables, and fruits. Though the population of the area is mainly youth similar to other regions of the country, data from the 2010 Ghana population and housing census indicate that the Eastern region is among the regions in the country with a high proportion of older adults. Approximately, 43% of the older population in Ghana live in three regions—the Ashanti region, the Greater Accra region, and the Eastern region [27]. The Ashanti and Greater Accra regions have been the sites for many research works on the older Ghanaian population, while few studies have been conducted in the Eastern region. This influenced the selection of the Eastern region of Ghana for the study. The Kwahu South District was then randomly chosen by the lottery method out of the 26 districts within the region.

### 2.2. Recruitment and Eligibility

Data were collected from 103 persons aged 65 and above in the Kwahu South District of Ghana. A multistage sampling approach (simple random and purposive) as demonstrated by Palinkas et al. [28] was adopted in selecting the respondents of the study. The district was divided into five clusters, of which one was randomly selected for the study using the lottery method for the study. Within the one cluster randomly selected, a total of 103 respondents were selected purposively to complete the administered questionnaires. This was adequate to produce robust estimates because Tabachnick and Fidell [29] had prescribed a minimum sample of 50 for computing robust estimates adequately. The purposive sampling was used to select persons within the age of interest and who are also of sound mind. The purpose of the study was explained to each potential participant and those who were of sound mind, willing, and could comprehend the issues that were selected to partake in the study. The elderly who were very ill, mentally unstable, and or had slurred speech were excluded from the study. Six of the participants did not provide enough information for the questionnaire administered, and thus, they were exempted from the analysis. Six other participants who were identified as key informants were selected purposively for a focus group discussion (FGD). The six people selected had lived at the study site for an average of 17 years and were therefore thought to be conversant with the challenges the aged like themselves face. Additionally, they comprised of a pastor, market queen, pensioneer, retired dietitian, retired district doctor, and a retired headmaster and by these characteristics, they are previewed to the challenges faced by the elderly within the population.

### 2.3. Data Collection Instruments

A structured questionnaire was used to collect the quantitative data. The questionnaire consisted of five distinct sections. The first section captured the sociodemographic data of participants, and the second part of the questionnaire consisted of a list of foods to which participants were to indicate which was most frequently consumed. The foods were categorized under four (4) food groups, namely, animal and animal products, cereals, vegetables, and fruits. This simple food frequency questionnaire was used to lessen the burden of recall. Data on the general health and nutritional challenges were collected in the third and fourth sections, respectively, while the last section was used to collect information on the determinants of food intake. These sections were made up of options to which participants were to indicate which frequently applied to them in a form of a Likert scale. The qualitative data were collected using an observational checklist and a focus group discussion guide. Instruments were validated by face, content, and construct after careful scrutiny by colleagues, research supervisors, and senior lecturers who are expects in food and nutrition research. After pilot testing and analyses, the instruments were found to be reliable, with a Cronbach alpha coefficient of 0.83.

### 2.4. Data Collection Procedure

Two trained research assistants were selected to assist with data collection based on their experience in nutrition research, aging issues, and communication skills. The questionnaire was interviewer-administered and the questions were translated verbatim into the local language of the participants. A focus group discussion guide was used to collect qualitative data on health and nutritional challenges. Participants were then asked questions to gain insights into the challenges they face in meeting their food needs. The FGD took approximately one hour to complete. Participants were assigned alphabets from A-F to keep the information obtained confidential. The session was both audio-taped and transcribed by the assistant moderator for the analyses and discussion.

### 2.5. Data Analysis

Quantitative data collected were analysed using Statistical Package for Social Sciences (SPSS) version 20. Descriptive statistics was used to analyse categorical data which are presented in frequencies and percentages. The qualitative data from the focus group discussion were transcribed verbatim from the tape recordings. The FGD was conducted in Twi which is the commonest local language in the study area and subsequently transcribed into English by research and language experts into electronic and printed forms for thematic analysis in order to minimize data loss. The transcribed data were read several times by researchers prior to data analysis. This was carried out to identify recurrent themes in the data. The themes were used to create a coding system including coding categories and a description of each category to guide the classification of responses to codes. The researcher and her two trained research assistants coded all transcripts, assigned coding categories to responses, and then evaluated all discrepancies in coding. Only minor discrepancies were observed, which were resolved by examining the original data and developing a consensus on interpretation. The qualitative software package CDC EZ-test version 3.10 C was used to facilitate data management and coding. Reliance on text-tagging software program helped to code and categorise responses in the original transcripts, thus providing a direct means by which emergent themes could be checked against and identified with the source material. Findings were presented as a description of recurrent themes, as well as a reporting of the frequencies of each code, using the FGD as the unit of analysis. Thus, the themes were developed inductively and explanatory accounts were developed in recursive engagement with the data set. Specifically, deviant cases or instances which did not conform to the accounts of the data were used to inform and amend these explanations [30, 31]. Extracts were not exclusively assigned to separate themes and the overlap between themes in the data was used to inform the broader analysis.

## 3. Results

### 3.1. Quantitative Data

#### 3.1.1. Sociodemographic Characteristics of Study Participants

Table 1 shows the sociodemographic characteristics of study participants. About 60% of the study participants were males, while the rest were females (39.2%). About half (44.3%) of the study participants were between the age of 65 and 69 years, and 30.9% were between the age of 60 and 64 years. Only 3.1% of participants were above the age of 75 years. About 40% of the participants were widowed, 33% were divorced, and 3.1% were cohabiting. More than half of the study participants had completed basic or secondary school. More than 50% of study participants were either retired, unemployed, casual workers, or part time workers.

#### 3.1.2. Dietary Habits of Study Participants

Table 2 shows the dietary habits of participants. Rice (34%) was the most frequently consumed cereal, followed by maize (22.6), wheat (19.6%), and sorghum (15.5%). Game (47.1%) was the most frequently consumed animal source food. This was followed by fish (24.9) and eggs (16.3). Meat was the least consumed (1.2%). Garden eggs (27.8%), leafy vegetables (22.4%), and tomatoes (21.7) were the most frequently consumed vegetables. Among the fruit group, banana (63.9%) and mango (19.6%) were the most frequently consumed.

#### 3.1.3. Determinants of Food Habits

The determinants of food habits among study participants were mood (41.2), stress (24.8%), and culture (23.3%).  Table 3 shows the determinants of food habits among study participants.

#### 3.1.4. General Life Challenges of the Elderly

Financial constraints (43.3%) were the challenge most indicated by participants followed by health challenges (25.8%). Loss of senses and mobility challenges were reported by 13.4% and 10.3% of participants, respectively.  Table 4 shows the general challenges of the elderly.

Table 5 presents the barriers reported to the attainment of food and nutrients needs. Medication and polypharmacy as well as mental and psychological problems were reported by 25.1% and 21.3% of participants, respectively. Technological (20.1%) and mobility (18.8%) challenges were also indicated.

### 3.2. Results from Focus Group Discussion

The focus group discussion (*n* = 6) was conducted to explore in-depth health and nutritional challenges of the elderly. Researchers analysed the focus group discussion around three coded themes: clinical and physical health status of the elderly, food habits (food choices and drivers of food choice), and nutrition knowledge.

### 3.3. Clinical and Physical Health Status of the Elderly

Participants asserted that health challenges such as poor vision, fatigue, toothache, and chronic knee, and joint pains affect their health and wellbeing, as well as the ability to obtain and cook their own foods. One participant explained it as captured as follows:

*“Constant toothache has made me to resort to the consumption of only soft and liquid foods though I prefer solid foods.”*

Another also described the situation as follows:

*“Until now, eating any food was optional but now eating hard foods and dairy products remain a luxury due to the loss of my teeth.”*

Participants also mentioned that they face a lot of physical difficulties that impairs their mobility. Some of the physical problems mentioned included hip degeneration and balance issues. One of the focus group members mentioned that bald hair has affected his physical comfort as an individual. Additionally, participants reported, they and a number of their age group have been clinically diagnosed with the following diseases; cardiovascular diseases (A, F, and E), prostate diseases (D), diabetes (A, B, C, and F), and hypertension (D, E, and F). Participants reported that clinical and physical changes evidently influenced their abilities to live independently, initiate and maintain physical activity, and socially engage. Additionally, participants hinted that such changes have led some of them to intensely use community health facilities and depend on others for survival. Participant E describes his situation as follows:

*“In an uncertain world where aging is certain, I sit idle and rely on the benevolence of others.”*

### 3.4. Food Habits

Participants were also asked to indicate which foods they often consumed. The foods that were frequently mentioned were game, fish, snails, and eggs. Of the fruits and vegetables groups, those that participants often consumed were banana, orange, pineapple, mango, avocado, okra, garden eggs, and cocoyam leaf (“kontomire”). Participants further explained that these food choices are driven by the taste and smell of the food, cost, their mood, culture, and appetite. Participant A made the following statement:

*“I prefer dried fish and boiled animal foods, slightly cooked and raw vegetables, squeezed and blended fruits, as well as boiled, pounded, and mashed roots and tubers.”*

Participants also affirmed that mental health challenges such as the feeling of apathy, phobic disorders, cognitive challenges, sexual dysfunction, loneliness, maltreatment, and feeling of neglect make them lose interest in shopping for food, food preparation, and eating. Respondents also mentioned financial constraints as a barrier to the attainment of their nutritional needs. This was captured from a participant as follows:

*“We know that fruits and vegetables are good for our health and can prolong our lifespan, but they are very expensive and as such we eat them as and when they are in season.”*

Another participant also explained it as follows:

*“To tell you the truth we often eat fruits only when we can afford or have them presented to us as gifts and/or when these fruits are in season. Honestly, this situation is scary yet it is the reality.”*

### 3.5. Nutrition Knowledge

Participants viewed fruits, vegetables, low-fat foods, and animal foods particularly game as healthy and vital for graceful greying. Fruit and vegetable consumption was frequently cited as being healthy, and a preference for vegetables over fruits was noted; many indicated this was due to the high cost and unavailability of fruits. Some participants noted that vegetables helped their vision and help built their resistance against diseases. There was consensus among participants that fat and oils found in red meats, butter, and whole milk must be avoided due to their adverse effects on human health. All participants were also made to mention the sources of their food and nutrition knowledge. The commonest sources cited were parents, health professionals, food labels, and the media. However, the vast majority of participants confirmed that parents and/or family played a crucial role in instilling knowledge of food habits in them. Beyond these, specific sources of nutrition knowledge identified by participants were media sources such as cooking shows on radio and television (A, B, and C), recipe books, magazines, and newsletters (A, B, and D), previous work experience in food-related jobs (D) and food labels (D, E, and F).

Participant B stated as follows:

*“I learnt about food preparation and its side effects extensively from my parents and female relatives at home.”*

Participant E captured it as follows:

*“I watched the TV cooking show “Onga Kitchen” and I started eating healthy.”*

Again, participant A reported what she heard on the radio:

*“Breakfast is the most crucial meal of the day so I make sure to prepare a “healthy breakfast” for my family to start the day.”*

Participants were of the view that nutrition knowledge helped them to eat healthy and live longer. Most participants emphasized that “healthy foods” protect them from illness, as well as prevent overweight and obesity and food-related diseases such as heart attack, diabetes, and hypertension. Focus group discussion members mentioned that processed and canned foods are unhealthy.

Participant D explained as follows:

*“When you eat something unhealthy, you become moody and tired because the fat gets retained in your body and fills your heart and clogs your blood vessels.”*

Participants further indicated that healthy eating helped them to gain more energy, stay younger, and improve their self-esteem, and they are able to focus on tasks with less stress. Related to this, some members said it was vital for them to eat healthy to serve as role models for other family members.

For example, participant F reported as follows:

*“I want to eat healthy so I become a role model for my children so they enjoy healthy food options.”*

## 4. Discussion

The main aim of this study was to assess the dietary habits of the aged and the nutritional challenges they face. Three main findings emerge from this study and they are discussed as follows.

Staple food consumption is high among study participants especially those foods that are common within the study area. For instance, game which was indicated as the frequently consumed animal source of food is not uncommon in the Eastern region [32]. Data from the Ministry of Agriculture also show that banana is the main fruit produced in the Kwahu South district [33]. Reliance on own food production is associated with low dietary diversity. There is research evidence to indicate that staple food consumption and consumption of own produced foods is common among the elderly and the financially deprived [34]. Though most staple foods tend to confer some health benefits because they are usually low in fat, they are also low in fruits and vegetables which are required for healthful aging [35]. In most African countries such as Ghana, staple or traditional foods consist of a high proportion of carbohydrate with a lesser quantity of vegetables which are mostly the cooked and small amount of protein [36]. Fruits and vegetables are amongst the most expensive group and may not be affordable for the elderly. As revealed by the sociodemographic data, the proportion of the aged who were divorced and or widowed was substantially high, a situation that can result in being economically disadvantaged. This is exacerbated by the fact that the majority of them were either unemployed, retired, casual, or part-time workers and as such face financial difficulties. Steiner-Asiedu et al. [37] in congruence documented that greater than 50% of the elderly participants in their study had to skip meals due to the lack of money. Furthermore, Blankson and Hall [38] found that being provided with financial support such as cash by children was associated with improved nutritional status.

Findings further revealed that mood, stress, and culture were the most selected predictors of food choice. Findings from a study conducted among women by Leeds et al. [39] found that mood was a strong predictor of food choice with the low mood being associated with unhealthy food consumption and vice versa. Bad mood and stress-related consumption is typically characterized by overeating, bingeing, and vomiting, which may have an adverse impact on the nutritional status of the elderly [40, 41]. Nutrition education, social support, and economic support should be implemented and strengthened to improve the social wellbeing and subsequently the nutritional status of the elderly. The Ghana Ghanaian government implemented the Livelihood Empowerment Against Poverty (LEAP) program in 2008 and it involved the provision of stipends to the extremely poor and elderly to support their basic needs, such as food. However, there are reports of some irregularities that render the program quite ineffective and thus limit its potential positive impact on the livelihoods of the elderly [42].

Additionally, the prevalence of physical and clinical challenges can affect the mood of the elderly and pose a nutritional challenge to the elderly by affecting their psychological and mental health and result in polypharmacy. Some medication may interfere with appetite and even nutrients consumed and as such have an impact on dietary habits and nutritional status. This deepens the need to implement preventive nutritional programmes at early stages that have the potential to avert the development of chronic diseases in old age. Tooth loss can result in dysphagia which seriously threaten the nutrition of the elderly [43].

Finally, resource constraints such as financial challenges can lead to poor nutritional choices even in the face of high nutritional knowledge. Price is one of the critical predictors of food choice and influences consumer purchasing and consumption behaviour [44]. Thus, providing adequate nutrition education through media platforms among others without an enabling environment does very little to solve the problem of unhealthy food consumption. Fiscal policies are needed to make healthy food affordable to the poor, rich, elderly, and young alike.

Longitudinal studies are needed to confirm the findings of this study and also provide compelling evidence on the nutritional status of the geriatric population, the nutritional challenges they face, and how improvement in their economic status impact their overall nutrition.

## 5. Conclusion

This study assessed the dietary habits and the nutritional challenges of the elderly in the Eastern region of Ghana. Data from the study show that there is a high consumption of staple foods among study participants especially those that are grown within the study area. Financial and polypharmacy, technological challenges, and toothache are prominent issues that affect the nutrition of the elderly. Additionally, despite high nutrition knowledge of the benefits of fruits and vegetables, financial constraints, and their high cost is a barrier to their consumption. Social interventions such as the LEAP programme should be expanded to provide food assistance consistently to the elderly. Social programmes are also required to ameliorate the feeling of loneliness, stress, and mood challenges.

## Figures and Tables

**Table 1:** Sociodemographic characteristics of study participants.

Demographic variables	*N* = 97	Frequency	Percent
Gender	Male	59	60.8
	Female	38	39.2

Age groupings	65–69	43	44.3
	60–64	30	30.9
	70–74	19	19.6
	75+	3	3.1
	Unknown	2	2.1

Marital status	Widowed	38	39.2
	Divorced	32	33.0
	Married	17	17.5
	Never married	7	7.2
	Cohabitation, etc	3	3.1

Educational background	O/A level	36	37.1
	Middle level	18	18.6
	Primary	15	15.4
	Postsecondary	13	13.4
	Professional	8	8.3
	Vocational/technical	3	3.1
	Tertiary	3	3.1
	No schooling	1	1.0

Employment status	Full-time	38	39.2
	Part-time	19	19.6
	Unemployed	16	16.5
	Retired	14	14.4
	Casual labour	8	8.2
	Others	2	2.1

**Table 2:** Dietary habits of respondents.

Food groups	Frequency	Percentage
*Cereals*		
Rice	33	34.0
Maize	22	22.6
Wheat	19	19.6
Sorghum	15	15.5
Millet	7	7.2
Others	1	1.2
*Animal source food*		
Game	46	47.1
Fish	24	24.9
Egg	16	16.3
Pork	8	8.1
Poultry	2	2.4
Meat	1	1.2
*Vegetables*		
Garden eggs	27	27.8
Leafy vegetables	22	22.4
Tomatoes	21	21.7
Okra	17	17.9
Others	10	10.2
*Fruits*		
Banana	63	63.9
Mango	10	19.6
Pawpaw	9	9.2
Orange	5	5.2
Others	2	2.1

**Table 3:** Determinants of food habits.

Determinants of food habits	Frequency	Percentage
Mood	40	41.2
Stress	24	24.8
Culture	21	21.3
Food preference	10	10.1
Others	2	2.6

**Table 4:** General challenges of the elderly.

Challenges *N* = 97	Frequency	Percentage
Finances	42	43.3
Health	25	25.8
Mobility	10	10.3
Loss of senses	13	13.4
Fear of death	7	7.2

**Table 5:** Nutritional challenges faced by respondents in meeting their food needs.

Nutritional challenges	Frequency^*∗*^	Percentage (%)
Medications and polypharmacy	80	25.1
Mental and psychological problems	68	21.3
Technological challenges	64	20.1
Mobility	60	18.8
Other age-related challenges	47	14.7
Total	319	100.0

^*∗*^Respondents were given the opportunity to give multiple responses to challenges.

## Data Availability

The data used to support the findings of this study can be made available by the corresponding author upon reasonable request.
